# The DnaJ-Like Zinc Finger Domain Protein PSA2 Affects Light Acclimation and Chloroplast Development in *Arabidopsis thaliana*

**DOI:** 10.3389/fpls.2016.00360

**Published:** 2016-03-24

**Authors:** Yan-Wen Wang, Si-Ming Chen, Wei-Jie Wang, Xing-Qi Huang, Chang-Fang Zhou, Zhong Zhuang, Shan Lu

**Affiliations:** State Key Laboratory of Pharmaceutical Biotechnology, School of Life Sciences, Nanjing UniversityNanjing, China

**Keywords:** *Arabidopsis thaliana*, DnaJ-like, zinc finger, chloroplast, thylakoid, xanthophyll, light acclimation

## Abstract

The biosynthesis of chlorophylls and carotenoids and the assembly of thylakoid membranes are critical for the photoautotrophic growth of plants. Different factors are involved in these two processes. In recent years, members of the DnaJ-like zinc finger domain proteins have been found to take part in the biogenesis and/or the maintenance of plastids. One member of this family of proteins, PSA2, was recently found to localize to the thylakoid lumen and regulate the accumulation of photosystem I. In this study, we report that the silencing of *PSA2* in *Arabidopsis thaliana* resulted in variegated leaves and retarded growth. Although both chlorophylls and total carotenoids decreased in the *psa2* mutant, violaxanthin, and zeaxanthin accumulated in the mutant seedlings grown under growth condition. Lower levels of non-photochemical quenching and electron transport rate were also found in the *psa2* mutant seedlings under growth condition compared with those of the wild-type plants, indicating an impaired capability to acclimate to normal light irradiance when *PSA2* was silenced. Moreover, we also observed an abnormal assembly of grana thylakoids and poorly developed stroma thylakoids in *psa2* chloroplasts. Taken together, our results demonstrate that PSA2 is a member of the DnaJ-like zinc finger domain protein family that affects light acclimation and chloroplast development.

## Introduction

The biosynthesis of chlorophylls and carotenoids and the assembly of thylakoid membranes are key processes for the development of plastids and are critical for the photoautotrophic growth of plants (Mochizuki et al., 1996; Pogson and Albrecht, 2011; Pogson et al., 2015). Different factors, including enzymes, transcription factors, structural proteins, and chaperones, are found to regulate the biogenesis and functioning of chloroplasts through these two processes under different conditions (Albrecht et al., 2006; Aluru et al., 2006; Sakamoto et al., 2008; Stephenson et al., 2009; Pfalz and Pfannschmidt, 2013; Zhong et al., 2013). Among these factors, the involvement of the DnaJ-like zinc finger domain proteins has been revealed only in recent years (Brutnell et al., 1999; Ham et al., 2006; Lu S. et al., 2006; Shimada et al., 2007; Lu Y. et al., 2011; Whippo et al., 2011; Zhong et al., 2013). These proteins are characterized by a C-terminal 4× repetition of the CxxCx(G)x(G) (the glycine residues are not absolutely conserved) motif resembling the zinc finger domain of bacterial DnaJ proteins, but do not retain the J-domain of those DnaJ proteins (Preisigmuller and Kindl, 1993).

Brutnell et al. (1999) characterized the first member of this family, BUNDLE SHEATH DEFECTIVE2 (BSD2). As a stroma protein, BSD2 is required for the accumulation of ribulose-1,5-bisphosphate carboxylase/oxygenase (Rubisco) in maize (Brutnell et al., 1999). Further studies have demonstrated that other DnaJ-like zinc finger domain proteins are also involved in the function and development of plastids. For examples, ORANGE (OR) from cauliflower and its orthologs from other plant species are located in both the chloroplast and the nucleus and regulate the transition from non-pigmented plastid forms into carotenoid-accumulated ones (Lu S. et al., 2006; Kim et al., 2013; Sun et al., 2015; Zhou et al., 2015). LOW QUANTUM YIELD OF PHOTOSYSTEM II1 (LQY1) is a thylakoid protein that functions in the repair and assembly of photosystem II (PSII) under high irradiance (Lu, 2011; Lu Y. et al., 2011). Moreover, in tobacco, Tsip1 is found to physically associate with the chloroplast surface and can be recruited to the nucleus to promote the expression of stress-related genes (Ham et al., 2006).

In a recent study, another DnaJ-like zinc finger domain protein, PSA2, was identified in both maize and *Arabidopsis thaliana* as a thylakoid lumen protein that promotes the biogenesis of photosystem I (PSI) (Fristedt et al., 2014). The silencing of *PSA2* resulted in severely retarded growth and pale green leaves, with increased photoinhibition due to the defect in PSI. The characterization of PSA2 provided novel insight to the function of the DnaJ-like zinc finger domain proteins in modulating the adaptation of chloroplasts to light stress.

In the present work, we studied the subcellular localization of PSA2, and compared the growth, pigment components, gene expression, chlorophyll fluorescence and chloroplast ultrastructure of the *psa2* mutant and the wild-type (WT) *A. thaliana* seedlings. Our results showed that both light acclimation and chloroplast development are affected by the silencing of *PSA2*.

## Materials and Methods

### Plant Materials and Growth Conditions

All *A. thaliana* plants, including the wild type (WT) and two mutants of *PSA2* (*At2g34860*), *psa2-1* (CS445540, i.e., GABI_475C12, with the T-DNA insertion at the 5′-UTR of *PSA2*) and *psa2-2* (CS430127, i.e., GABI_314G07, with T-DNA insertion in the second intron), are of the Col-0 ecotype background. Seeds purchased from the *Arabidopsis* Biological Resource Center (ABRC, Ohio State University, Columbus, OH) were surface-sterilized and then plated onto Murashige and Skoog (MS) medium. After 3 d stratification at 4°C in the dark, seeds were germinated at 22–25°C under a light intensity of 100 μmol photons m^-2^ s^-1^ with a 14 h/10 h light/dark photoperiod. Two-week-old seedlings were moved to grow in soil (a mixture of peat moss, vermiculite and perlite at 1:1:1) under the same conditions.

### Molecular Manipulation and Plant Transformation

Genomic DNA was extracted from mature leaves (Murray and Thompson, 1980). Homozygous seedlings of the *psa2-1* mutant and its progenies were screened according to the SIGnAL iSect tool^1^ using three primers, i.e., a forward primer, LP, that binds from -610 bp upstream of the translation initiation codon (ATG) of *PSA2*, a reverse primer, RP, that binds from 300 bp downstream of the ATG codon, and the primer 8409 targets the sequence of vector pAC106 that was originally used for generating the T-DNA insertion mutants. All primers used for polymerase chain reaction (PCR) in this study are listed in Supplement Table S1. Genomic DNA extracted from each individual seedling was used as a template. Homozygous progeny lines were used in this study.

Total RNA was isolated using the RNAiso Plus reagent (TaKaRa, Shiga, Japan) and was then reverse transcribed to a first strand cDNA pool using a PrimeScript 1st Strand cDNA Synthesis Kit (TaKaRa) according to the manufacturer’s manuals. Gene expression was analyzed by quantitative real-time PCR (qPCR) in a Thermal Cycler Dice Real Time System TP800 (TaKaRa) using SYBR Premix ExTaq II (TaKaRa), following the manufacturer’s instructions, and calculated according to the comparative *C*_T_ method (Schmittgen and Livak, 2008). For each sample, at least three biological replicates were analyzed, and each experiment was repeated three times. *ACT2* (*At3g18780*) was used as a reference.

For the expression of a fusion protein of PSA2 with a C-terminal green fluorescent protein (GFP), the full-length open reading frame (ORF) of *PSA2* was amplified from the first stand cDNA pool by primers PAS2-GFP-F and PSA2-GFP-R. The amplicon was electrophoresed, extracted from the gel using a SanPrep Column DNA Gel Extraction Kit (Sangon, Shanghai, China), digested with *Nco*I and *Spe*I (TaKaRa), and ligated into pCAMBIA1302 (CAMBIA, Canberra, Australia) that was digested with the same enzymes and purified. This generated the construct *35S:PSA2-GFP* for the expression of the PSA2-GFP fusion protein by protoplast transfection according to Yoo et al. (2007).

We also amplified a genomic DNA fragment ranging from -2,000 bp upstream of the translation initiation codon to 1,000 bp downstream the stop codon of *PSA2*, using primers PSA2-FL-F and PSA2-FL-R with genomic DNA as a template. The amplicon was electrophoresed, extracted from the gel, digested with *Kpn*I and *Sal*I (TaKaRa), and ligated into pCAMBIA1300 (CAMBIA) that was digested and purified as described above. This generated the construct *PSA2:PSA2* for genetic complementation. The construct was transferred into the *Agrobacterium tumefaciens* strain GV3101 by electroporation (Lu S. et al., 2006) for transformation of *psa2-1* plants using the floral dip method (Clough and Bent, 1998).

For all PCR amplifications, high-fidelity PrimeStar DNA polymerase (TaKaRa) was used according to the manufacturer’s instruction throughout this study.

### Pigment Analysis

Pigments were extracted from the leaves and analyzed by reverse-phase high-performance liquid chromatography (HPLC) using a Waters 2695 separation module with a Spherisorb ODS2 column (5 μm, 4.6 mm × 250 mm) and 2998 photodiode array detector (PDA) (Waters, Milford, MA) following the method of Li et al. (2001). Deuteroporphyrin was used as an internal standard.

### Chlorophyll Fluorescence Measurements

Chlorophyll fluorescence was measured using a portable MINI-PAM fluorometer (Walz, Effelrich, Germany). Before the measurements, leaves were dark-adapted for 20 min. The minimal yield of fluorescence (*F_o_*) under measuring light (650 nm) was measured at a very low light intensity (0.8 μmol photons m^-2^ s^-1^). To estimate the maximum fluorescence yield (*F_m_*), a saturating pulse (0.8 s, 5,000 μmol photons m^-2^ s^-1^) was applied. The ratio *F_v_*/*F_m_* = (*F_m_–F_o_*)/*F_m_* was calculated to indicate the maximum photochemical efficiency (Maxwell and Johnson, 2000). For the measurement of light-response curves of non-photochemical quenching (NPQ), electron transport rate (ETR) and PSII quantum yield (ΦPSII), dark-adapted plants were illuminated at a series of photosynthetically active photon flux densities (PPFD) (0, 46, 96, 168, 257, 358, 535, 731, and 1,107 μmol photons m^-2^ s^-1^). The duration of illumination at each light intensity was 3 min, after which a saturation pulse was applied.

### Chloroplast Isolation, Fractionation, and Western Blot Analysis

The isolation of intact chloroplasts and the preparation of stroma and thylakoid protein fractions were performed according to Hall et al. (2011). Fractionation of grana core, grana margin, and stroma lamellae was performed as described by Lu Y. et al. (2011).

Each fraction was suspended in sodium dodecyl sulfate (SDS) loading buffer (Green and Sambrook, 2012), denatured, and resolved by SDS-polyacrylamid gel electrophoresis (SDS-PAGE). Proteins were semi-dry transferred to nitrocellulose membrane Protran BA 83 (GE Healthcare, Pittsburgh, PA, USA) for immunodetection. A peptide (CGLPNNKGLLRRPGA) was synthesized based on the putative amino acid sequence of PSA2 and used as an antigen to immunize rabbits by GenScript (Nanjing, China). Antibodies against LHCB2, RbcL and PsaA were purchased from Agrisera (Vännäs, Sweden). Horseradish peroxidase (HRP)-conjugated secondary antibody against rabbit IgG was from Promega (Madison, WI). Immobilon Western HRP substrate for chemiluminescent detection was from EMD Millipore (Darmstadt, Germany). Common protocols (Green and Sambrook, 2012) and the manufacturer’s manuals for SDS-PAGE, semi-dry blotting and Western detection with chemiluminescent substrate were followed.

### Microscopy

The *35S:PSA2-GFP*-transfected protoplasts were cultivated in the dark for 12 h and then observed using a FLUOVIEW FV1000 Laser Confocal Microscopy System (Olympus, Tokyo, Japan) (Lee and Hwang, 2011). For transmission electron microscopy (TEM) analysis of chloroplast ultrastructure, leaves from 4-week-old seedlings were fixed, embedded and sectioned according to Faso et al. (2009). A Hitachi-7700 transmission electron microscope was used for observation and image capturing.

### Sequence Analysis

The TAIR10 dataset of *A*. *thaliana* proteins was scanned using PatMatch^2^ for all proteins with the 4× repeats of the CxxCx(G)x(G) motifs (with at least one glycine residue in each motif). Proteins with this repeat at their C-termini were manually identified. Sequences of DnaJ-like zinc finger domain proteins from other plant species were downloaded from GenBank. Sequences were aligned using the ClustalX program and manually edited.

### Statistical Analysis

Statistical significance was tested using GraphPad Prism6 (GraphPad Software). Data are shown as the mean ± SE of at least three replications.

## Results

### PSA2 Belongs to the DnaJ-Like Zinc Finger Domain Protein Family

**FIGURE 1:**
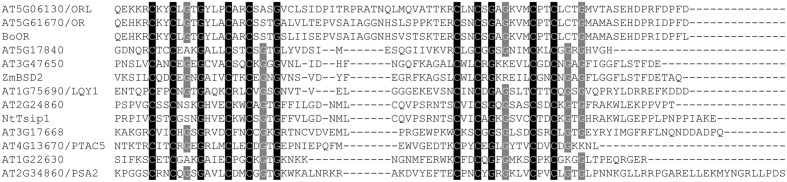
**Alignment of the C-terminal regions of DnaJ-like zinc finger domain proteins in *Arabidopsis thaliana*.** Previously reported members from other plant species, including BSD2 from maize (ZmBSD2), OR from cauliflower (BoOR) and Tsip1 from tobacco (NtTsip1) were also incorporated. Sequences were aligned using ClustalX and manually edited. Conserved amino acid residues of the zinc finger domain were shaded.

Because DnaJ-like zinc finger domain proteins from different plants are found to regulate the development and function of plastids (Brutnell et al., 1999; Ham et al., 2006; Lu S. et al., 2006; Shimada et al., 2007; Lu Y. et al., 2011; Whippo et al., 2011; Zhong et al., 2013), we scanned the TAIR10 dataset of *A. thaliana* proteins, and identified a total of 10 homolog proteins that possess the 4× repeats of the CxxCx(G)x(G) motifs (with at least one glycine residue in each motif) at their C-terminal regions. Sequence comparison revealed that, in each protein, the four CxxCx(G)x(G) motifs are grouped into two pairs. In each pair, two motifs are separated by three amino acid residues which are not conserved. Six of these proteins (or their orthologs in other plant species) have been previously identified to have a chloroplast localization, including OR (At5g61670) (Lu S. et al., 2006), PSA2 (At2g34860) (Fristedt et al., 2014), LQY1 (At1g75690) (Lu, 2011; Lu Y. et al., 2011), BSD2 (At5g17840) (Brutnell et al., 1999), Tsip1 (At2g28460) (Ham et al., 2006), and pTAC5 (At4g13670) (Zhong et al., 2013) (**Figure 1**).

### *psa2* Mutants Showed Pigment-Defective and Growth-Retarded Phenotypes

**FIGURE 2:**
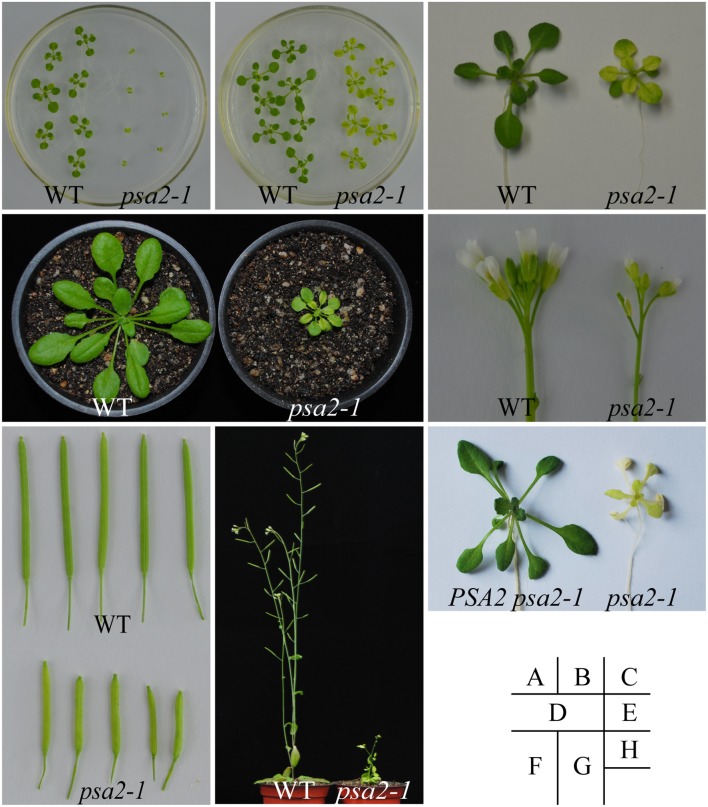
**Phenotype of the *psa2-1* mutant plants. (A,B)** *psa2-1* seedlings germinated on MS plates without **(A)** or with **(B)** 1% sucrose for 2 weeks. **(C)** *psa2-1* seedlings grown in soil for an additional 2 weeks after germination on MS plates. **(D)** A representative *psa2-1* seedling grown in soil for 4 weeks after germination on MS plates. **(E–G)** Inflorescence **(E)**, siliques **(F)**, and mature plant **(G)** of the *psa2-1* mutant. **(H)** Transformation with a wild type *PSA2* gene fragment complemented the growth of *psa2-1*. Seedlings germinated on MS plates for 2 weeks were photographed.

We ordered T-DNA insertion lines of these ten genes encoding DnaJ-like zinc finger domain proteins from ABRC. For each gene, when available, at least three lines with different T-DNA insertion positions were used in this study. We first germinated all these mutant lines on MS plates without sucrose for two weeks. For most of these lines, juvenile seedlings of the mutants did not show a distinct phenotype from the WT plants (data not shown). However, for *PSA2*, seedlings of the *psa2-1* line with the T-DNA insertion at its 5′-UTR showed pale yellow leaves and severely retarded growth (**Figure 2A**). Another line, *psa2-2*, with the T-DNA insertion in the second intron died shortly after de-etiolation (data not shown). The expression level of *PSA2* in *psa2-1* was about the 17% of the WT level, whereas in *psa2-2*, it was undetectable (data not shown). When we then germinated these mutant lines on MS plates supplied with 1% sucrose, the *psa2-1* seedlings showed variegated leaves and partially promoted growth (**Figure 2B**), whereas the *psa2-2* line did not show significant recovery (data not shown). In the variegated leaves of the *psa2-1* mutant, only the vein areas were green and other regions were pale yellow (**Figure 2C**). When we moved these *psa2* mutant seedlings from MS plates with sucrose to the soil, only the *psa2-1* plants survived, although they still had variegated leaves and retarded growth (**Figure 2D**). We repeated this for three batches, and observed identical results for each mutant line.

When flowering, the *psa2-1* seedlings were much smaller than the WT plants (**Figure 2G**) and had thinner inflorescence branches and fewer flowers (**Figure 2E**). The juvenile siliques of the *psa2-1* mutant were shorter than those of the WT plants, and they also had a pale yellow color (**Figure 2F**). All *psa2-1* mutant seedlings had similar phenotypes at each developmental stage, and only representative pictures are presented in **Figure 2**.

We then selfed the *psa2-1* line and harvested the F_1_ seeds. After germination, no phenotypic segregation was observed in the progeny population. All mature F_1_ plants showed variegated leaves, consistent with their parents. We randomly selected ten different F_1_ plants, and our PCR analysis showed that all of these were homozygous (data not shown). We used a genetic fragment from –2,000 bp upstream of the translation initiation codon to 1,000 bp downstream of the stop codon of *PSA2* (*PSA2:PSA2*) to transform the *psa2-1* plants. The transgenic progeny grew normally with green leaves, similar to the wild-type (**Figure 2H**). This demonstrated that the disruption of the *PSA2* gene is responsible for the observed phenotypes of the *psa2* mutants.

### Tissue Specificity of the Expression of *PSA2*

**FIGURE 3:**
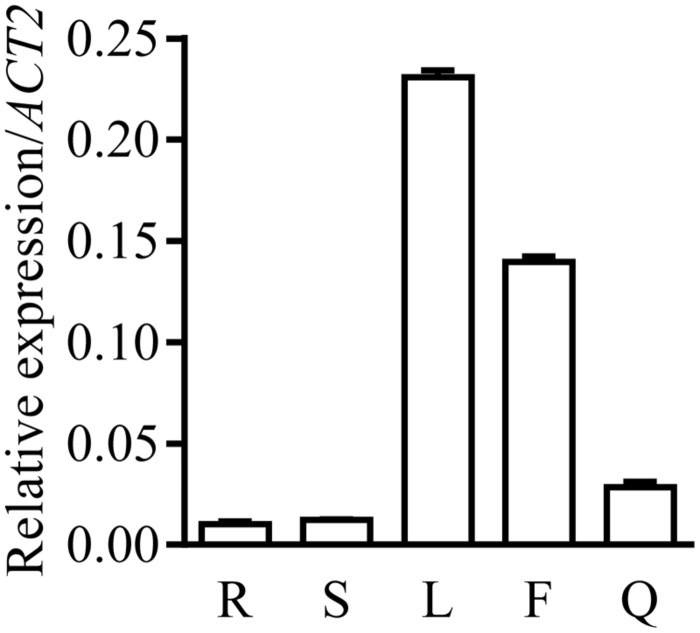
**Tissue specific expression of *PSA2* in *Arabidopsis thaliana*.** Transcript levels of *PSA2* in roots (R), stems (S), rosette leaves (L), flowers (F), and siliques (Q) determined by quantitative real-time PCR are expressed relative to *ACT2* (*At3g18780*). Data represent means ± SE (*n* = 6).

We compared the expression levels of *PSA2* in different tissues. From our qPCR results, *PSA2* was highly expressed in leaves, followed by flowers and siliques. The expression of *PSA2* in both root and stems was relatively low (**Figure 3**).

### Subcellular Localization of PSA2

**FIGURE 4:**
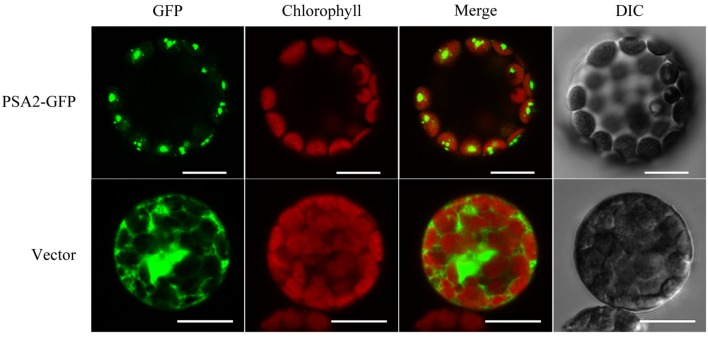
**Subcellular localization of PSA2 in chloroplasts.** Protoplasts transformed with *35S:PSA2-GFP* were observed under a FLUOVIEW FV1000 Laser Confocal Microscopy System (Olympus). Those transformed with pCAMBIA1302 empty vector were observed as a control. Bar = 20 μm.

TargetP^3^ predicted a chloroplast localization of PSA2. This was confirmed by our protoplast transformation and confocal observation. Transient expression of the PSA2-GFP fusion protein showed a clear fluorescence signal in chloroplasts (**Figure 4**).

**FIGURE 5:**
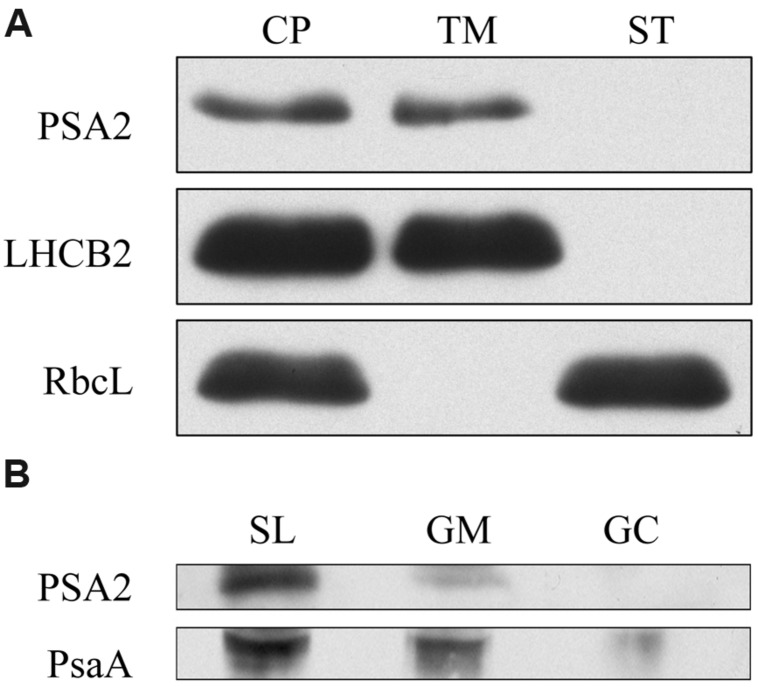
**Western blot showing that PSA2 localizes predominantly in stroma lamellae. (A)** Purified chloroplasts (CP) were separated into the thylakoid membrane (TM) and stroma (ST) fractions, separated by SDS-PAGE, blotted and probed with the antiserum against PSA2. LHCB2 and RbcL were probed as controls. **(B)** Thylakoid membranes were furtherly subfractionated into stroma lamellae (SL), grana margin (GM), and grana core (GC) fractions, separated by SDS-PAGE, blotted, and probed with the antiserum against PSA2. PsaA was probed as a control.

For a detailed understanding of PSA2 localization, we isolated intact chloroplasts from mature leaves of the WT plants and further lysed and centrifuged them to separate the supernatant (containing the outer and inner envelope membranes and stroma) and the pellet (containing mainly thylakoids). Our Western blots showed that PSA2 was only present in the pellet fraction, suggesting a thylakoid localization (**Figure 5A**). Furthermore, we separated the thylakoids into stroma lamellae and grana margin and grana core fractions by differential ultracentrifugation. Our Western blot results showed that PSA2 co-localized with PsaA, a component of the PSI reaction center, which is predominantly localized in SL fraction and could also be detected in grana margin (**Figure 5B**).

### *psa2* Seedlings Accumulated Abnormal Levels of Chlorophylls and Carotenoids

**FIGURE 6:**
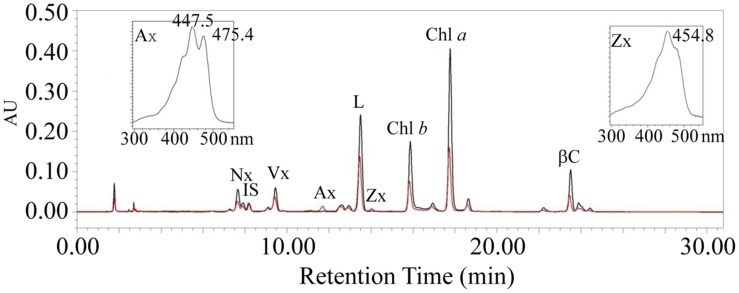
**High-performance liquid chromatography (HPLC) analysis of pigments in rosette leaves of the *psa2-1* mutant (red) and the wild-type plants (black).** Peaks are neoxanthin (Nx), internal standard (IS), violaxanthin (Vx), antheraxanthin (Ax), lutein (L), zeaxanthin (Zx), β-carotene (βC), and chlorophylls (Chl *a* and Chl *b*). Insets are spectra profiles of Ax and Zx.

Because the *psa2-1* leaves showed a variegated phenotype, we quantified the pigment contents in rosette leaves of 4-week-old seedlings by HPLC (**Figure 6**).

The amounts of different pigments are listed in **Table 1**. The chl *a* and *b* contents in *psa2-1* were only 37 and 45% of the WT level, respectively. This resulted in a decrease of the chl *a*/*b* ratio from 2.68 to 2.21. The lutein, β-carotene, violaxanthin (Vx), and neoxanthin (Nx) contents in the *psa2-1* mutant were also lowered to approximately 30-40% of their corresponding WT levels. However, two carotenoids belonging to the xanthophyll cycle, zeaxanthin (Zx), and antheraxanthin (Ax), were found to accumulate in *psa2-1* leaves but were not detectable in WT leaves. Overall, *psa2-1* had only 41.29% carotenoids compared with the WT plants.

**Table 1:** Leaf pigment profile of the *Arabidopsis thaliana* wild-type (WT) and *psa2-1* mutant plants (μg/g fresh weight).

	WT	*psa2-1*
Chl *a*	1036.60 ± 85.44	381.42 ± 102.57**
Chl *b*	486.60 ± 35.15	216.96 ± 49.00**
Chl *a*/*b*	2.14 ± 0.31	1.75 ± 0.09**
		
Lutein	270.80 ± 6.33	111.97 ± 1.88**
β-carotene	97.73 ± 7.66	29.70 ± 2.36**
Zeaxanthin	0	8.23 ± 2.66**
Antheraxanthin	0	8.42 ± 1.44**
Violaxanthin	53.55 ± 1.63	19.47 ± 0.64**
Neoxanthin	58.92 ± 2.36	17.21 ± 0.27**

Data represent means ± SE (Student’s *t* test; *n* = 6. **P < 0.01).

**FIGURE 7:**
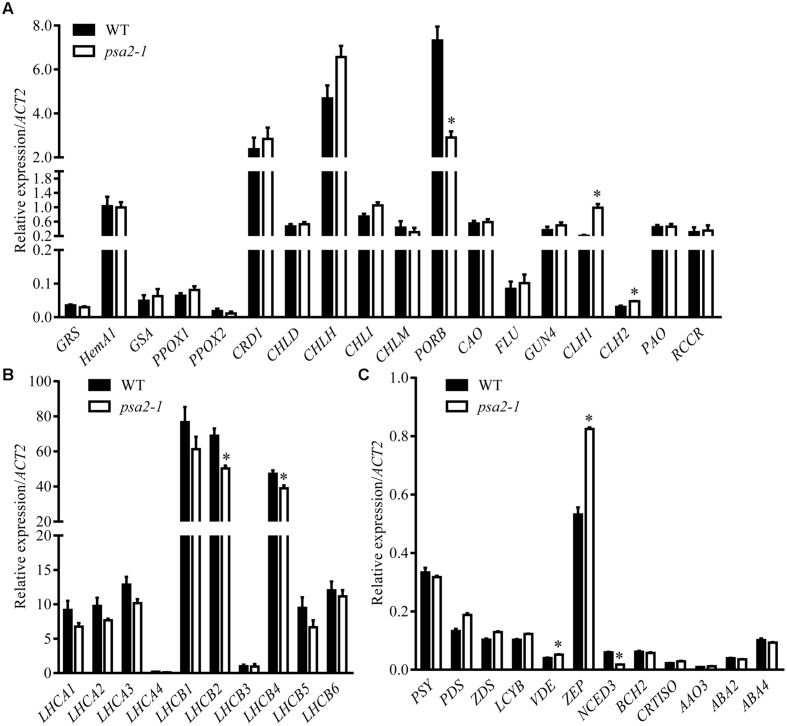
**Expression of the genes encoding enzymes for chlorophyll metabolism **(A)**, chlorophyll binding proteins **(B)** and enzymes for carotenoid metabolism **(C)** in rosette leaves of the 4-week-old *psa2-1* and wild-type (WT) seedlings.** Transcript abundance of each gene was quantified by quantitative real-time PCR and are expressed relative to *ACT2* (*At3g18780*). Data represent means ± SE (Student’s *t* test; *n* = 6. *P < 0.05).

Because chlorophylls and total carotenoids were significantly lower in the *psa2-1* mutant, we determined the expression levels of genes in the chlorophyll and carotenoid metabolic pathways. Our results demonstrated that *PROTOCHLOROPHYLLIDE OXIDOREDUCTASE B* (*PORB*), a key gene in chlorophyll biosynthesis, was downregulated in the *psa2-1* mutant to 39.7% of the WT level (**Figure 7A**), whereas two genes for chlorophyll turnover, *CHLOROPHYLLASE1* and *2* (*CLH1* and *CLH2*), were upregulated to 4.76- and 1.55-fold of the WT levels, respectively (**Figure 7A**). We further quantified the expression of genes for chlorophyll-binding proteins (*LHCA* and *LHCB*), only *LHCB2* and *LHCB4* were significantly repressed in the *psa2-1* mutant (**Figure 7B**).

For carotenoid metabolism, we did not observe a significant variation in the expression of most of the genes. *ZEAXANTHIN EPOXIDASE* (*ZEP*) and *VIOLAXANTHIN DE-EPOXIDASE* (*VDE*), two genes in the xanthophyll cycle, showed slight but significant upregulation to 1.41- and 1.24-fold, respectively, of the WT levels (**Figure 7C**). The expression of *NINE-CIS-EPOXYCAROTENOID DIOXYGENASE 3* (*NCED3*), the gene for carotenoid turnover, was downregulated to 29% of the WT level in the *psa2-1* mutant (**Figure 7C**).

### PSA2 is Required for Chloroplast Development

**FIGURE 8:**
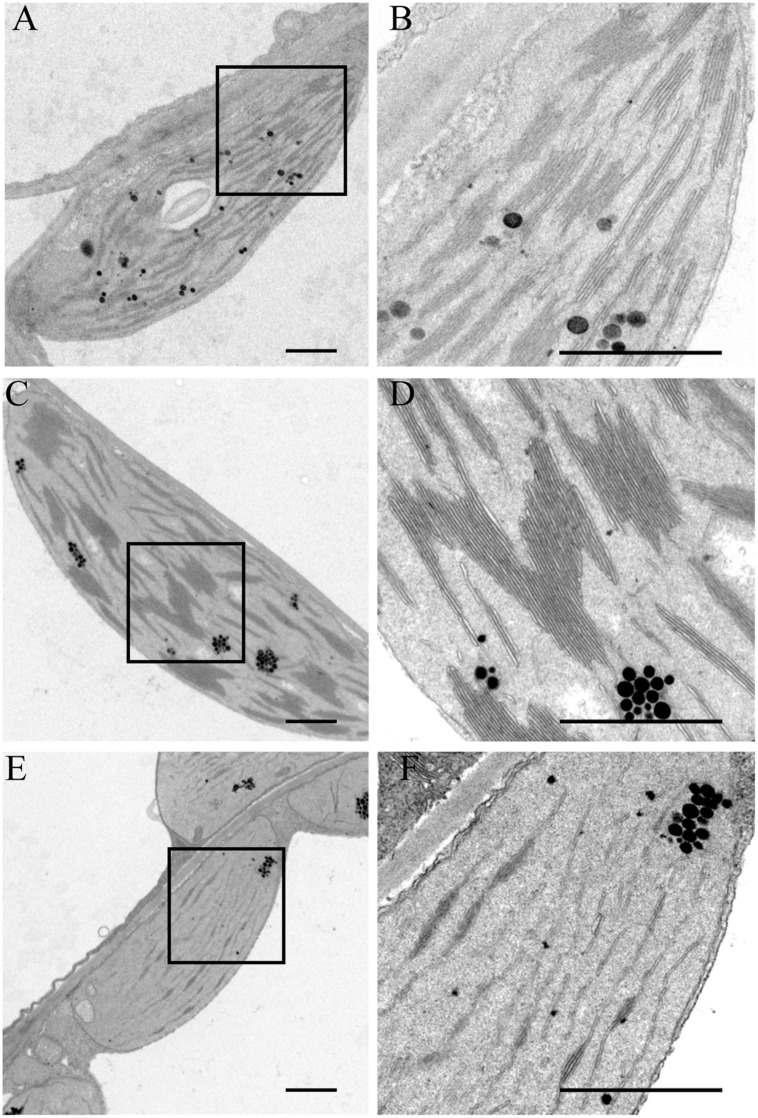
**Transmission electron microscopy (TEM) of chloroplasts from leaves of the wild-type plants **(A,B)**, and from the green **(C,D)** and pale yellow **(E,F)** regions of the variegated leaves of the *psa2-1* seedlings.** Bar = 1 μm. **(B,D,F)** are magnified view of the boxed regions in **(A,C,E)**, respectively.

The variegated leaves of the *psa2-1* mutant suggested a possible defect in chloroplast development. To this end, we sectioned leaves of the 4-week-old seedlings and observed them with TEM. In WT chloroplasts, approximately 10 layers of thylakoids were stacked in each grana, which were connected to each other by stroma lamellae. In each chloroplast, only a few starch grains and plastoglobules were observed (**Figures 8A,B**). In the green regions of the variegated *psa2-1* leaves, approximately 30–50 layers of thylakoids were slantly piled and connected to other stacks from the margin. No distinct stroma lamellae were observed (**Figures 8C,D**). Dozens of plastoglobules were found in each chloroplast in this region (**Figures 8C,D**). In the pale yellow regions of the mutant leaves, almost no thylakoid stacking was found in the chloroplast, and the stroma lamellae were abnormally assembled (**Figures 8E,F**).

### *psa2* Mutants are Defective in Acclimating to Normal Growth Light

To assess the impact of the low accumulation of photosynthetic and photoprotective pigments and the poor chloroplast development on photosynthetic activity, we performed non-invasive fluorometric analyses. Compared with the WT plants, the *psa2-1* mutant had a significantly lower minimal fluorescence (*F_o_*), whereas its maximal fluorescence (*F_m_*) was similar to the WT level. This resulted in a significant reduction in the ratio of viable fluorescence (*F_v_* = *F_m_*–*F_o_*) to *F_m_*, which is an indicator of the efficiency of PSII photochemistry, from 0.832 in the WT plant to 0.672 in *psa2-1* (**Table 2**).

**Table 2:** Chlorophyll fluorescence parameters in the leaves of the wild-type (WT) and *psa2-1* plants grown under growth light (100 μmol photons m^-2^ s–^1^).

	WT	*psa2-1*
*F_o_*	0.194 ± 0.006	0.382 ± 0.060**
*F_m_*	1.156 ± 0.021	1.167 ± 0.204
*F_v_*/*F_m_*	0.832 ± 0.007	0.672 ± 0.023**

Data represent means ± SE (Student’s *t* test; *n* = 6. **P < 0.01).

**FIGURE 9:**
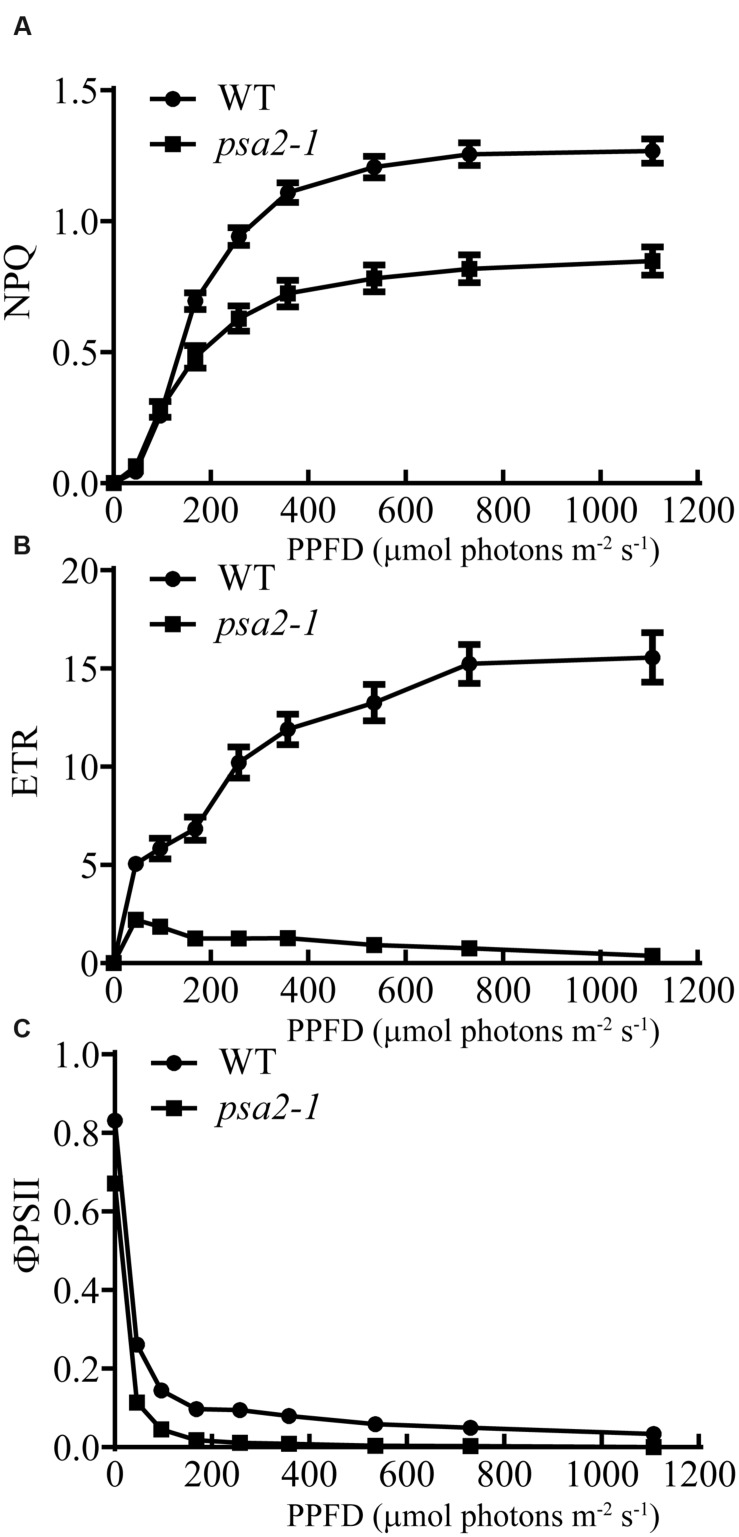
**Light-responsive curves of the non-photochemical quenching (NPQ) **(A)**, photosynthetic electron transport rate (ETR) **(B)** and PSII quantum yield (ΦPSII) **(C)** in the wild-type (WT) and *psa2-1* seedlings.** The measurements were taken at the following photosynthetically active photon flux densities (PPFD): 0, 46, 96, 168, 257, 358, 535, 731, and 1,107 μmol photons m^-2^ s^-1^. Data represent means ± SE (*n* = 6).

We then measured the light-responsive curves to assess NPQ, ETR and ΦPSII. At 96 μmol photons m^-2^ s^-1^, a light intensity close to the growth condition, NPQ of the *psa2-1* plants was similar to that of the WT plants. However, above this light intensity, NPQ of the *psa2-1* plants dropped significantly from its corresponding WT level (**Figure 9A**). Although the ETR of the WT plants increased with the increasing of actinic light intensity in our measurements, that of the *psa2-1* seedlings dropped immediately when the light intensity was greater than 46 μmol photons m^-2^ s^-1^. For example, when the light intensity reached 96 μmol photons m^-2^ s^-1^, the ETR of *psa2-1* was only 31.71% of the level in the WT plants (**Figure 9B**). The ΦPSII of *psa2-1* was always lower than that of the WT plants at all light intensities we measured, e.g., it was approximately 31.57% of the WT level at 96 μmol photons m^-2^ s^-1^ and was almost abolished when the light intensity was greater than 196 μmol photons m^-2^ s^-1^ (**Figure 9C**).

## Discussion

Previously, Fristedt et al. (2014) reported that PSA2 interacts with a protein complex that contains PsaG, and the *psa2* mutants specifically lost the PSI core complex. In our study, we confirmed the *PSA2*-silencing phenotypes (retarded growth and pale yellow leaves) reported by Fristedt et al. (2014). However, when we supplied sucrose to the medium, the vein areas of the juvenile *psa2-1* mutant leaves turned green, showing a promoted development of chloroplasts comparing with the pale yellow regions. Because the mutant line with the T-DNA insertion in the intron (*psa2-2*) was lethal when grown in soil, as previously reported and also found in our study, our work focused on the *psa2-1* mutant as well.

In addition to the previously reported phenotypes, we observed an abnormal accumulation of Zx and Ax in leaves of the *psa2-1* mutant. In plants, ZEP catalyzes the conversion from Zx to Ax and then to Vx in the light, and VDE catalyzes the reverse reaction in the dark. The conversions among these three carotenoid species form the xanthophyll cycle, which protects the photosynthetic apparatus (Demmig-Adams and Adams, 1996). Although Zx and Ax are usually undetectable in plants growing under normal growth light, they are found to accumulate under high light irradiance that inhibits ZEP but induces VDE (Chen and Gallie, 2012). The accumulation of Zx and Ax in *psa2-1* plants suggests that the mutant is unable to acclimate to normal growth light. Moreover, *psa2-1* had a higher *F_o_* and a lower *F_v_*/*F_m_* compared with the WT plants, suggesting the occurrence of photoinhibitory damage under growth light. Because PSA2 is found to regulate the accumulation of PSI, the reduction of *F_v_*/*F_m_* in *psa2-1* is similar to the *ppd1* mutant, which also has impaired PSI biogenesis (Liu et al., 2012).

The activation of VDE and the impaired NPQ suggest a lower trans-thylakoid ΔpH and an inhibited electron transport downstream of PSII, similar to the *ppd1* mutant (Maxwell and Johnson, 2000; Liu et al., 2012). This was confirmed by our determination of the ETR and ΦPSII under a series of light intensities. The ETR of the *psa2-1* plant dropped immediately when the light intensity was above 46 μmol photons m^-2^ s^-1^. Considering that the *psa2-1* mutant cannot effectively accumulate PSI, a lower ETR suggests that the photosynthetic electrons produced by PSII under growth light were not well accepted by PSI in *psa2-1*. As a result, ΦPSII in *psa2-1* also dropped rapidly and was approximately 0 under light intensities above 257 μmol photons m^-2^ s^-1^, while the WT plants still retained substantial activity. These results are typical for mutants blocked in photosynthetic electron transport downstream of PSII, such as *ppd1* (Meurer et al., 1996; Liu et al., 2012).

Besides the results of the carotenoid quantification and chlorophyll fluorescence, which support the conclusion of Fristedt et al. (2014), an important finding in our work is that PSA2 is essential for the assembly of thylakoid membranes.

Previous studies showed that the impairment of photosystems usually occurs with alternations in the chloroplast ultrastructure (Rock et al., 1992; Goss et al., 2007). For example, grana stacking in the thylakoid membranes is very important for the process of overall NPQ, and it has been reported that partial unstacking of the grana membranes strongly reduces the capacity for heat dissipation (Goss et al., 2007). In *psa2-1*, grana thylakoids were not observed in the pale yellow regions of the variegated leaves. Although there are more layers of thylakoids stacked in each grana in chloroplasts in the green regions, this may be a compensation for the defective organization of the macrostructure, which has yet to be determined but may be a result of the defective accumulation of PSI. A distinct difference between the ultrastructures of the WT and *psa2-1* chloroplasts is the lack of inter-grana stroma lamellae.

In chloroplasts, PSII and light harvesting complex II are almost exclusively found in the stacked regions of the grana membranes, whereas PSI and light harvesting complex I are located in the stroma thylakoid membranes and are also found in the margin regions of the grana thylakoids (Goss et al., 2007). Our Western blot results showed that PSA2 co-localizes with PsaA, a component of PSI, in stroma thylakoid and, to a lesser degree, in grana margin. This subcellular localization of PSA2 helps to explain the function of this protein. Similar to PPD1, which also localizes to the same fraction and interacts with PsaA/PsaB of PSI to regulate the assembly of PSI (Goss et al., 2007), PSA2 may be involved in assembly of the thylakoids through its protein-disulfide isomerase activity; however, its mechanism remains unclear.

Taken together, the spectroscopy measurements, biochemical analyses and chloroplast ultrastructure observations demonstrate the impairments in the function of PSI and the assembly of thylakoid membranes in the *psa2-1* mutant, thus providing a straightforward explanation for the pigment-deficient and growth-retarded phenotypes of this mutant. This work showed that PSA2 is a member of the DnaJ-like zinc finger domain protein family that is involved in the regulation of the function and development of the chloroplast.

## Author Contributions

Y-WW, S-MC, W-JW, X-QH, and ZZ performed experiments. C-FZ, ZZ, X-QH, and SL analyzed the data. S-MC and SL wrote the manuscript.

## Conflict of Interest Statement

The authors declare that the research was conducted in the absence of any commercial or financial relationships that could be construed as a potential conflict of interest.

## Footnotes

1 http://signal.salk.edu/tdnaprimers.2.html

2 https://www.arabidopsis.org/cgi-bin/patmatch/nph-patmatch.pl

3 http://www.cbs.dtu.dk/services/TargetP/
