# Supplementary material for: The DnaJ-Like Zinc Finger Domain Protein PSA2 Affects Light Acclimation and Chloroplast Development in Arabidopsis thaliana
Source: Front Plant Sci. 2016 Mar 24;7:360. doi: 10.3389/fpls.2016.00360 (PMC4806229; doi:10.3389/fpls.2016.00360)
Supplement: Supplementary file 1 [file Table_1.PDF]

Supplement Table S1. Primers used in this study.

Primer	Sequence (5’-3’, restriction sites are underlined)
For vector construction	
PAS2-GFP-F	CCATGCCATGGCGGCATCATCATCTCA
PSA2-GFP-R	GACTAGTTGAATCAGGAAGAAGCCGACC
PSA2-FL-F	TTTGGTACCGTTCACTTCGAGAAAACTGAAAG
PSA2-FL-R	TTTGTCGACTTTTTGCTTATATGCTTTTGTGTCTG
For homozygous seedling selection	
LP	GATCAAGAAAATTAAAGCAACCGACAGTTCCATGC
RP	GGACTTTAACACACAGAACCAACAAGAATGTAAGG
8409	ATATTGACCATCATACTCATTGC
For quantifying gene expression by qPCR	
ACT2	AAGGCCGAAAATAAAGTTGTAAGAGATAAACCCGC<br>GAGCGGAAGAAGATGAGATTGAGGAAGATTCA
PSA2	CGCAGACTGAAAAAAATGGCGGCATC<br>TGAGGAAGAATCCGAATCGTTGGAGTCG
LHCA1	GTCGTATCAGAATGGCTGCTCACTGGATGC<br>GCCCATCTACAGTGGATGAGCTCTGACTCTTTGTA
LHCA2	GTGCTTCTTCTGCCATCGCTGCCAT<br>TGCTTCCCGGGAACCAGATTGGTCTATCT
LHCA3	TCTCTTGGTGACAGAAGAAAAGAGTTGAGGAACAG<br>GCTTTAACAACAAAGGAACTCTTCTTCTGTGAGGC
LHCA4	ACAAAATCCCCTCCAAGTCTTATTTCTTCACAACC<br>GCCGAGGCATGAGTAGTGACAGTAGCCATTAT
LHCB1	AGGCCTTCGCTGAGTTGAAGG<br>GCCTCTACAACGGAGTGAACC
LHCB2	CATTGAAGGCTACAGAATCGGAGG<br>CTCACCTGCATCTGTGAAACAAAATC
LHCB3	GCATCAACGGTCTTGATGGTGTTG<br>GACTTCTTCTGTGCATAGCAAATAGAAG
LHCB4	GTTCCTGAGTGGTACGATGCTG<br>GCCAACCTCCCGTTTGCTAG
LHCB5	CACCAACGGATTGGATTTCGAGG<br>GTCAAAAGGAGTTCCAATCGAACAAAG
LHCB6	AGCAAACGATGGGTCGATTTCTTC<br>TATCTACAGAATCAAAAACATCATGCCG
GRS	CTGTGTGCCCTAGACACAC<br>GAAGCCATGTCAGCTTCAGC
HemA1	GTTGGATCTTGTGTTGGTGAAG<br>CTGTTCACGATACCTCGGC
GSA	CTGGATTTCGTCTAGCCTATGG<br>TGCGAAAAAGAATCCAAACATACCG
PPOX1	CTATCTCGTACCCGAAAGAAGC<br>GGAATGGCTTGAGGCCATAC
PPOX2	CTCTCGGTTTTAATCACCACATTC

	CAACGGGAATGCTTTCCTCC
CRD1	CCGAGAAAATCGGGTACTGG
	TTCGGTTTGTCTCGATGATGAC
CHLD	GGAGCCCTGGTTATCTTTGTG
	GGCTCGACCATCGGTTATC
CHLH	CAGGACATGTACTTGAGCCG
	GTACCACTTTGGATTCAGCAGC
CHLI	CGGAAGAAGGAGAGCTTAGG
	GCTGCAAGTGCTTTTGCTGC
CHLM	GAACCGGTTTGCTCTCGATTC
	GCTCTCCAATTCTCTTTAAGATATCAT
porA	GAACTCCGATTATCCATCAAAACG
	GAGGAATATGCTCTCTGAACAAAC
porB	CCTTCAAAGCGTCTCATCATCG
	GGCACGGAAGAGAGGAATGT
porC	CAAAACGTATGATCATCGTAGGATC
	AAGCCGAAACAGCGGTATGTG
CAO	TGGATTTGGCCCGGTGATG
	TCTAGTTTCCCGGGTTTTGATATC
FLU	GTCTTATGCACCAAGCTTGAGTTAT
	GTGATTCCAGAATCTTCACTTTCC
GUN4	TCCGAGGTGAAAACAATCTCCC
	CCACTGTTATCATCAGCTGTCG
CLH1	GATCCAGTCGCAGGAACTAAC
	CCACCTACAAAGCTCCTCATC
CLH2	GGTACTCCTCGAATCTAAAGATC
	CAATAAGAACTCTTCCCTCTAATCC
PAO	GAAAGACCCGTTCCATCGTTTG
	TGGAAGGGAGAGGTTGGTTAG
RCCR	GTGGTCGAACTAATACAGAGC
	CTTCTTCTTCCTTCACACACC
PSY	GGGTTGCTACTTCTTCTCTAAATCCAGACCCAATG
	TCGGTTCCTTACAAAAGAAGAACTCCAAGTTGGTA
PDS	GGAGTACTGCTGGTCCTTTGCAGGTAGTTTGTG
	CCAGCACCAGCAATTACAACTTTCAAAGGCTT
ZDS	TAACAGAGCGATTGCTCTTATCTCAGTTGATTGGC
	GCGTTAACACTCATGTCGGAAACATCGGAG
LYC	TCTTTCTGGGAAGAAGATAATTGTCTCCATCTCCA
	CCCCCAAAATCGAAGAAAACACCAAAAGGATA
VDE	TTCTCCGGCTCTGTCTCTGTCTCTCTTCTTCC
	GCCAAGCCTACCAATACCATCATCACTTGAGAAA
ZEP	GTTTTCCGGAAAACCCGGAGGAGTATCTG
	CCACCTCCGGCAACTAAAACCCTCGATT
NCED3	GAAAAATGGCTTCTTTCACGGCAACGG
	AACATTGAGCTTACGTGTGACACGACTGGC
BCH2	TCTTTCTCCGCAAACCACCCTATATCCACC
	TGCTTTCTGGTTTGTTGTCATCGTCCATG
CRTISO	GAAGCTGCCTCGTATGGTTACAGTGAAATCCG
	ACAGCTAGCTGAGTCGCAGCAACTAATCCAC

AAO3	ATCCATCAACGACCTTACTTGAGTTCTTGCGA<br>TTCGTGTTTCCAAGACCTTCAGATGTAGTAATGGA
ABA2	GGAGGAGCCACAGGGATAGGTGAGAGCAT<br>CCATGGATGAAAAAAGCCGTCTCCTTGGAC
ABA4	ATTAGATTAGATCATCGTTGGAGCTTCATTGGAGG<br>CGGTAGTTCCAACCGCAAATACACTGCTTG
